# Clinicopathological and surgical comparisons of differentiated thyroid cancer between China and the USA: A multicentered hospital-based study

**DOI:** 10.3389/fpubh.2022.974359

**Published:** 2022-09-28

**Authors:** Juan Zhu, Kexin Sun, Jian Wang, Yutong He, Daojuan Li, Shuzheng Liu, Yunchao Huang, Min Zhang, Bingbing Song, Xianzhen Liao, He Liang, Qian Zhang, Mumu Shi, Lanwei Guo, Yongchun Zhou, Yanping Lin, Yanni Lu, Jiyu Tuo, Yafen Xia, Huixin Sun, Haifan Xiao, Yong Ji, Ci Yan, Jinwan Qiao, Hongmei Zeng, Rongshou Zheng, Siwei Zhang, Shaoyan Liu, Sheng Chang, Wenqiang Wei

**Affiliations:** ^1^Office of National Central Cancer Registry, National Cancer Center/National Clinical Research Center for Cancer/Cancer Hospital, Chinese Academy of Medical Sciences and Peking Union Medical College, Beijing, China; ^2^Department of Cancer Prevention, Institute of Cancer and Basic Medicine (IBMC), The Cancer Hospital of the University of Chinese Academy of Sciences (Zhejiang Cancer Hospital), Chinese Academy of Sciences, Hangzhou, China; ^3^Department of Head and Neck Surgery, National Cancer Center/National Clinical Research Center for Cancer/Cancer Hospital, Chinese Academy of Medical Sciences and Peking Union Medical College, Beijing, China; ^4^Cancer Institute, The Fourth Hospital of Hebei Medical University, Shijiazhuang, China; ^5^Henan Cancer Prevention and Control Office, The Affiliated Cancer Hospital of Zhengzhou University, Henan Cancer Hospital, Zhengzhou, China; ^6^Office of Yunnan Cancer Center, Yunnan Cancer Hospital, Kunming, China; ^7^Office of Cancer Prevention and Treatment, Hubei Cancer Hospital, Wuhan, China; ^8^Heilongjiang Cancer Center, Institute of Cancer Prevention and Treatment, Harbin Medical University, Harbin, China; ^9^Department of Cancer Prevention and Control, Hunan Cancer Hospital and the Affiliated Cancer Hospital of Xiangya School of Medicine, Central South University, Changsha, China; ^10^Scientific Research Education Department, National Cancer Center/National Clinical Research Center for Cancer/Cancer Hospital and Shenzhen Hospital, Chinese Academy of Medical Sciences and Peking Union Medical College, Shenzhen, China; ^11^Information Management and Big Data Center, The Tumor Hospital Affiliated to Xinjiang Medical University, Ürümqi, China; ^12^Science and Education Department, The Fifth People's Hospital of Qinghai, Xining, China; ^13^Medical Department, National Cancer Center/National Clinical Research Center for Cancer/Cancer Hospital and Shenzhen Hospital, Chinese Academy of Medical Sciences and Peking Union Medical College, Shenzhen, China; ^14^Human Resources Office, National Cancer Center/National Clinical Research Center for Cancer/Cancer Hospital, Chinese Academy of Medical Sciences and Peking Union Medical College, Beijing, China

**Keywords:** thyroid cancer, differentiated thyroid carcinoma, stage, surgery, lobectomy, total thyroidectomy, BMI, China

## Abstract

**Background:**

Thyroid cancer (TC), was the fastest-rising tumor of all malignancies in the world and China, predominantly differentiated thyroid cancer (DTC). However, evidence on TC stage distribution and influencing factors of late-stage were limited in China.

**Methods:**

We carried out a retrospective study and enrolled TC patients who were first diagnosed and hospitalized in 8 hospitals in China in 2017. Logistic regression was used to evaluate associations between influencing factors and DTC stage. We extracted eligible primary DTC records newly diagnosed in 2017 from the USA's Surveillance, Epidemiology, and End Results (SEER) database. We compared clinicopathological features and surgical treatment between our DTC records and those from the SEER database.

**Results:**

A total of 1970 eligible patients were included, with 1861 DTC patients with known stage. Among patients ≥45 years old, males (OR = 1.76, 95%CI 1.17–2.65) and those with new rural cooperative medical scheme insurance (NCMS) (OR = 1.99, 95%CI 1.38–2.88) had higher risks of late-stage DTC (stage III-IV). Compared with SEER database, over-diagnosis is more common in China [more DTC patients with onset age< 45 years old (50.3 vs. 40.7%, *P* < 0.001), with early-stage (81.2 vs. 76.0%, *P* < 0.001), and with tumors<2cm (74.9 vs. 63.7%, *P* < 0.001)]. Compared with the USA, TC treatment is more conservative in China. The proportion of lobectomy in our database was significantly higher than that in the SEER database (41.3 vs. 17.0%, *P* < 0.001).

**Conclusions:**

Unique risk factors are found to be associated with late-stage DTC in China. The differences in the aspect of clinicopathological features and surgical approaches between China and the USA indicate that potential over-diagnosis and over-surgery exist, and disparities on surgery extent may need further consideration. The findings provided references for other countries with similar patterns.

## Background

Thyroid cancer (TC) is the most common endocrine and head and neck malignancy worldwide. According to Global Cancer Statistics 2020, 586,202 new cases and 44,000 deaths occurred worldwide in 2020 (1). TC incidence has been rising at the fastest speed, predominantly differentiated thyroid carcinoma (DTC) (2, 3), and has already become a huge health concern in the current era. The incidence rate of TC has been increasing rapidly in China in the past decades. According to the national report released by the National Cancer Center in China, TC became the fastest-growing malignant tumor in China (4–7). Despite the slow progression for TC, gaps in 5-year survival exist between China and developed countries such as the USA (84.30 vs. 98.16%) (8, 9). Compared with advanced TC patients, early-stage patients are more likely to be cured and have a better prognosis (10–12). The stage information in the USA was collected in the Surveillance, Epidemiology, and End Results (SEER) database. Identifying stage distribution and influencing factors is beneficial to narrow the survival gap and improve the prognosis of TC. However, so far, evidence on TC stage distribution and influencing factors of late-stage were limited in China.

Because of the increasing prevalence of excessive BMI around the world, the impact of obesity on cancer risk has been extensively studied. Epidemiological evidence confirmed that BMI was independently associated with an increased incidence of DTC (13). However, the possible associations between BMI and aggressive clinicopathologic features of thyroid cancer have remained controversial. Several studies showed that Obesity was associated with several poor clinicopathologic prognostic features: extrathyroidal extension, multifocality, and advanced tumor/node/metastasis stage (14). On the contrary, others showed that there was no independent association between obesity and more aggressive clinicopathological features of TC (15). Previous related studies in China were limited to small samples from one or a small number of hospitals, and evidence at the population-based level was not available. Therefore, based on the multicenter, large-scale, population-based study in China, we further explored the associations between BMI and aggressive clinicopathological characteristics.

What's more, the reasons for the differences in TC disease burden may lie in the differences in cancer screening policies and clinical guidelines between China and the USA, which can be reflected by comparing clinicopathological features and therapeutic schedules between the two countries. However, few multicentered and large-scale studies focus on stage distribution, clinicopathological characteristics and surgical treatment of TC in China. To fill the evidence gap, the National Cancer Center in China initiated a multicenter hospital-based retrospective survey in 2017. We aimed (1) to ascertain DTC stage distribution at diagnosis and investigate relevant risk factors for late-stage DTC patients, and (2) to compare clinicopathological disparities and surgical treatments of DTC between our data and SEER data.

## Methods

### Study design and participants

**Figure 1:**
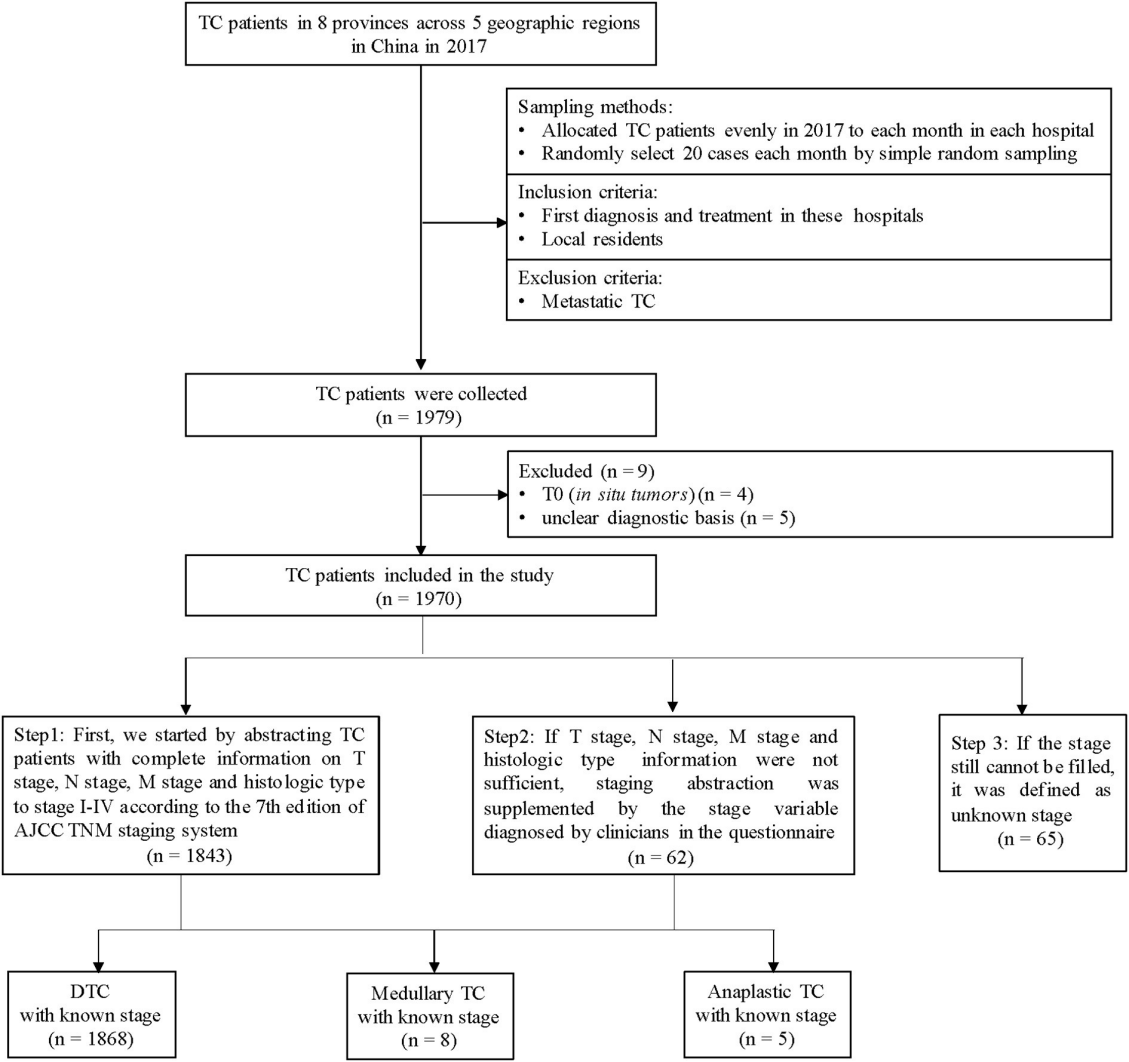
Flowchart of the study.

We carried out a retrospective investigation and included TC patients from 8 grade A tertiary hospitals in 8 provinces (Henan, Hubei, Shenzhen, Hunan, Heilongjiang, Xinjiang, Yunnan and Qinghai) in China. Inclusion criteria: (a) newly diagnosed as TC in 2017; (b) hospitalized between January to December in 2017; (c) resident of the province where the hospital was located. We excluded patients with other primary tumors that metastasized to the thyroid. The study flowchart was shown in Figure 1. Considering the unknown risks in the research process, the total target sample size of TC was determined as 1,920 cases (240 cases in each hospital). We allocated sample sizes evenly to each month, which was 20 cases per month for each hospital. We sorted all eligible TC cases according to discharge time in each month and selected the cases *via* simple random sampling.

The third edition of the International Classification of Diseases for Oncology (ICD-O-3) topography was used for identifying TC (C73.9). Trained investigators extracted information using a case report form from electronic medical records, including (1) demographic characteristics; (2) clinicopathological characteristics; and (3) therapeutic information.

### Staging

First, we started by abstracting TC patients with complete information on T stage, N stage, M stage and histologic type to stage I-IV according to the 7th edition of the American Joint Committee on Cancer (AJCC) Tumor-Node-Metastasis (TNM) staging system (16). If the above information (T, N, M and histologic type) were not sufficient, staging abstraction was supplemented by the stage variable diagnosed by clinicians in the questionnaire. If the stage still cannot be filled, it was defined as an unknown stage (Figure 1). Stage I and II were defined as early-stage, and stage III and IV were defined as late-stage.

### SEER database in the USA

SEER^*^Stat was used to generate a case listing. We extracted eligible primary TC records diagnosed in 2017 from the SEER database, collecting information including age, sex, histologic type ICD-O-3, derived SEER combined T, derived SEER combined N, derived SEER combined M, derived SEER combined stage (the 7th edition AJCC), tumor size summary, focality, surgery primary site, reason no cancer-directed surgery. A total of 13,053 TC cases in 2017 were downloaded from the SEER database. In order to be comparable with the subjects included in this study, we only included DTC patients with a known stage in the SEER database (*n* = 11,006).

### Statistical analysis

We used the Chi-square test or Fisher's exact test to compare the stage distribution, clinicopathological characteristics and therapeutic information among different groups. We estimated adjusted odds ratios (ORs) for late-stage DTC patients using logistic regression. In model 1, only sex at diagnosis was adjusted. In model 2, area and medical insurance were added. NCMS is a new community-based rural health insurance system now covering over 98% of rural residents in China, first implemented in 2003. In model 3, multiple factors including BMI, smoking, drinking, history of thyroid diseases and family history of TC were included by the enter method. In model 4, the main model, we used the stepwise method to identify significant variables. No collinear among variables was found in logistic regression. Data management, programming, and analyses were carried out using SAS 9.4. All tests of significance were two-tailed, and *P* < 0.05 was considered statistically significant.

## Results

### The risk factors of late-stage DTC patients

#### Characteristics of DTC patients by stage distribution

A total of 1,970 eligible patients were included, with a mean age of 44.3 ± 11.5 years, of which 96.7% (*n* = 1905) had known stage (Figure 1; Supplementary Table S1). 94.8% of TC with the known stage was DTC (*n* = 1868), and the characteristics of DTC patients were shown in Table 1. The proportion of DTC patients in urban areas, with urban insurance, was higher than patients living in rural areas and those with NCMS (52.9 vs. 47.1%, 31.6 vs. 23.2%). The female/male sex ratio was 3.3. As shown in Table 1, the proportion of early-stage and late-stage of DTC were 81.2 and 18.8%. Significant differences in stage distribution were found among different regions (*P* < 0.001). The characteristics of DTC patients by stage distribution, by age were presented in Supplementary Table S2.

**Table 1:** Characteristics of DTC patients by stage distribution.

**Characteristics**	**All**	**Stage I**	**Stage II**	**Stage III**	**Stage IV**	***P-*value**	**Early-stage (I/II)**	**Late-stage (III/IV)**	***P-*value**
	***n* (%)**	***n* (%)**	***n* (%)**	***n* (%)**	***n* (%)**		***n* (%)**	***n* (%)**	
N	1,868 (100.0)	1,465 (78.4)	52 (2.8)	220 (11.8)	131 (7.0)		1,517 (81.2)	351 (18.8)	
Area ^a^						< 0.001			< 0.001
Urban	988 (52.9)	811 (55.4)	25 (48.1)	102 (46.4)	50 (38.2)		836 (55.1)	152 (43.3)	
Rural	880 (47.1)	654 (44.6)	27 (51.9)	118 (53.6)	81 (61.8)		681 (44.9)	199 (56.7)	
Sex						0.011			0.068
Male	431 (23.1)	330 (22.5)	7 (13.5)	50 (22.7)	44 (33.6)		337 (22.2)	94 (26.8)	
Female	1,437 (76.9)	1135 (77.5)	45 (86.5)	170 (77.3)	87 (66.4)		1,180 (77.8)	257 (73.2)	
BMI ^b^, kg/m2						0.197			0.135
< 25.0	941 (66.0)	765 (67.3)	23 (56.1)	103 (60.9)	50 (64.1)		788 (66.9)	153 (61.9)	
≥25.0	484 (34.0)	372 (32.7)	18 (43.9)	66 (39.1)	28 (35.9)		390 (33.1)	94 (38.1)	
Medical insurance ^c^						< 0.001			< 0.001
Urban medical insurance	577 (31.6)	485 (33.9)	11 (21.2)	64 (29.5)	17 (13.3)		496 (33.5)	81 (23.5)	
NCMS	423 (23.2)	299 (20.9)	12 (23.1)	62 (28.6)	50 (39.1)		311 (21.0)	112 (32.5)	
Others	826 (45.2)	645 (45.1)	29 (55.8)	91 (41.9)	61 (47.7)		674 (45.5)	152 (44.1)	
Smoking history ^d^						0.144			0.378
Never	1,679 (90.4)	1,317 (90.4)	51 (98.1)	199 (90.5)	112 (86.8)		1,368 (90.7)	311 (89.1)	
Ever	179 (9.6)	140 (9.6)	1 (1.9)	21 (9.5)	17 (13.2)		141 (9.3)	38 (10.9)	
Drinking history ^e^						0.142			0.283
Never	1,747 (94.5)	1,372 (94.6)	52 (100.0)	206 (94.5)	117 (91.4)		1,424 (94.8)	323 (93.4)	
Ever	101 (5.5)	78 (5.4)	0 (0.0)	12 (5.5)	11 (8.6)		78 (5.2)	23 (6.6)	
History of thyroid diseases ^f^						0.441			0.256
Absent	1,657 (88.9)	1,302 (89.1)	49 (94.2)	191 (86.8)	115 (87.8)		1,351 (89.3)	306 (87.2)	
Present	207 (11.1)	159 (10.9)	3 (5.8)	29 (13.2)	16 (12.2)		162 (10.7)	45 (12.8)	
Family history of thyroid cancer						0.482			1.000
Absent	1,823 (99.0)	1,434 (99.0)	50 (98.0)	213 (99.5)	126 (98.4)		1,484 (98.9)	339 (99.1)	
Present	19 (1.0)	15 (1.0)	1 (2.0)	1 (0.5)	2 (1.6)		16 (1.1)	3 (0.9)	

^a^Using the area classification of the National Bureau of Statistics in China, we categorized each patient's place of residence into urban/rural.

^b^Body mass index (BMI) was calculated by the weight (kg) divided by the square of height (m).

^c^Medical insurance: Urban medical insurance, including urban employment-based basic medical insurance and urban resident-based basic medical insurance; NCMS, new rural cooperative medical scheme insurance; Others, including uninsured, other insurance and unknown.

^d^Smoking history: Ever, smoking at least 100 cigarettes in his or her lifetime.

^e^Drinking history: Ever, drinking at least 25g ethanol per week and last at least for 1 year.

^f^History of thyroid disease included thyroiditis, nodular thyroid, thyroid adenoma and thyroid capsule.

#### Multivariate analysis for late-stage DTC patients

Considering only 1.0% of DTC patients aged <45y were stage II, the multivariate analysis for late-stage was performed in DTC patients aged ≥45y (Table 2). The sex-adjusted ORs between late-stage and rural area, NCMS, BMI ≥ 25.0, smoking, drinking, history of thyroid diseases and family history of TC were 1.33 (1.02–1.74), 2.08 (1.44–2.99), 0.93 (0.67–1.30), 0.81 (0.50–1.34), 1.06 (0.57–2.00), 1.10 (0.74–1.65) and 1.21 (0.27–5.50), respectively. Male patients (1.76, 95%CI 1.17–2.65, *P* = 0.006) and those with new rural cooperative medical scheme insurance (NCMS) (OR = 1.99, 95%CI 1.38–2.88, *P* < 0.001) had a higher risk of late-stage DTC, after adjusting for area, BMI, smoking history, drinking history, history of thyroid disease and family history of TC.

**Table 2:** Adjusted ORs (95% CIs) for late-stage DTC patients aged ≥ 45y.

	**Model 1**		**Model 2**		**Model 3**		**Model 4**	
	**OR (95% CI)**	***P* value**	**OR (95% CI)**	***P* value**	**OR (95% CI)**	***P* value**	**OR (95% CI)**	***P* value**
**Sex**
Male	–	–	–	–	1.84 (1.15–2.94)	0.011	1.76 (1.17–2.65)	0.006
Female	–	–	–	–	Ref		Ref	
**Area**
Urban	Ref		–	–	Ref		–	–
Rural	1.33 (1.02–1.74)	0.037	–	–	0.90 (0.59–1.37)	0.624	–	–
**Medical insurance**
Urban medical insurance	Ref		–	–	Ref		Ref	
NCMS	2.08 (1.44–2.99)	< 0.001	–	–	2.43 (1.43–4.13)	0.001	2.20 (1.43–3.38)	< 0.001
**BMI, kg/m** ^**2**^
< 25.0	Ref		Ref		Ref		–	–
≥25.0	0.93 (0.67–1.30)	0.686	0.99 (0.71–1.39)	0.945	0.91 (0.64–1.29)	0.597	–	–
**Smoking history**
Never	Ref		Ref		Ref		–	–
Ever	0.81 (0.50–1.34)	0.417	0.84 (0.51–1.39)	0.497	0.89 (0.45–1.74)	0.734	–	–
**Drinking history**
Never	Ref		Ref		Ref		–	–
Ever	1.06 (0.57–2.00)	0.848	1.12 (0.59–2.14)	0.734	0.96 (0.38–2.43)	0.930	–	–
**History of thyroid diseases**
Absent	Ref		Ref		Ref		–	–
Present	1.10 (0.74–1.65)	0.632	1.10 (0.72–1.67)	0.655	0.72 (0.41–1.26)	0.249	–	–
**Family history of thyroid cancer**
Absent	Ref		Ref		Ref		–	–
Present	1.21 (0.27–5.50)	0.802	1.51 (0.33–6.87)	0.593	1.34 (0.22–8.33)	0.752	–	–

Model 1: adjusted sex.

Model 2: adjusted sex + area + medical insurance.

Model 3: sex, area, medical insurance, BMI, smoking history, drinking history, history of thyroid disease and family history of cancer (enter).

Model 4: sex, area, medical insurance, BMI, smoking history, drinking history, history of thyroid disease and family history of cancer (stepwise).

#### The association between BMI and aggressive clinicopathological features of DTC patients

As shown in Supplementary Table S3 and Table 3, compared with underweight and normal DTC patients, we found no evidences that overweight and obese was associated with tumor size (OR = 1.07, 95%CI 0.80–1.41, *P* = 0.655), pathologic T4 stage (OR = 1.30, 95%CI 0.72–2.38, *P* = 0.387), lymph node metastasis (OR = 0.89, 95%CI 0.70–1.14, *P* = 0.366), distant metastasis (OR = 0.70, 95%CI 0.22–2.24, *P* = 0.552) and late-stage (OR = 0.97, 95%CI 0.70–1.34, *P* = 0.838). Similar patterns were shown in the subgroup analysis stratified by age and sex.

**Table 3:** The association between BMI and aggressive clinicopathological features of DTC patients.

	**BMI**	**Tumor size (>2cm)**	**T stage (T4)**	**N stage (N1)**	**M stage (M1)**	**Late-stage (III/IV)**
		**OR (95% CI)**	**OR (95% CI)**	**OR (95% CI)**	**OR (95% CI)**	**OR (95% CI)**
All*	< 25.0	Ref				
	≥25.0	1.07 (0.80–1.41)	1.30 (0.72–2.38)	0.89 (0.70–1.14)	0.70 (0.22–2.24)	0.97 (0.70–1.34)
Age < 45**	< 25.0	Ref				
	≥25.0	0.91 (0.61–1.37)	1.50 (0.58–3.88)	0.77 (0.55–1.09)	0.80 (0.15–4.19)	–
Age ≥ 45**	< 25.0	Ref				
	≥25.0	1.26 (0.84–1.88)	1.20 (0.55–2.60)	1.04 (0.74–1.47)	0.66 (0.13–3.30)	0.99 (0.71–1.39)
Male***	< 25.0	Ref				
	≥25.0	0.94 (0.56–1.60)	2.20 (0.74–6.50)	1.11 (0.68–1.80)	1.10 (0.07–17.98)	1.39 (0.68–2.84)
Female***	< 25.0	Ref				
	≥25.0	1.14 (0.82–1.60)	1.02 (0.48–2.21)	0.84 (0.63–1.12)	0.62 (0.17–2.25)	0.86 (0.58–1.27)

^*^Adjusted for age at diagnosis, sex and area.

^**^Adjusted for sex and area.

^***^Adjusted for age at diagnosis and area.

### Comparations of clinicopathological features of DTC patients between the study and SEER database

As shown in Supplementary Table S4, compared with the 7th edition TNM staging system, we found the 8th staging downstages many DTC patients. 78.4 and 2.8% of DTC patients were distributed in stage I and stage II by the 7th edition TNM staging system. In contrast, 92.9 and 4.9% of DTC patients were distributed in stage I and stage II with the 8th edition staging. The dominant early-stage features were more obvious when we tried the 8th edition TNM staging (increased by 16.6%, *P* < 0.001) (Supplementary Table S4).

**Figure 2:**
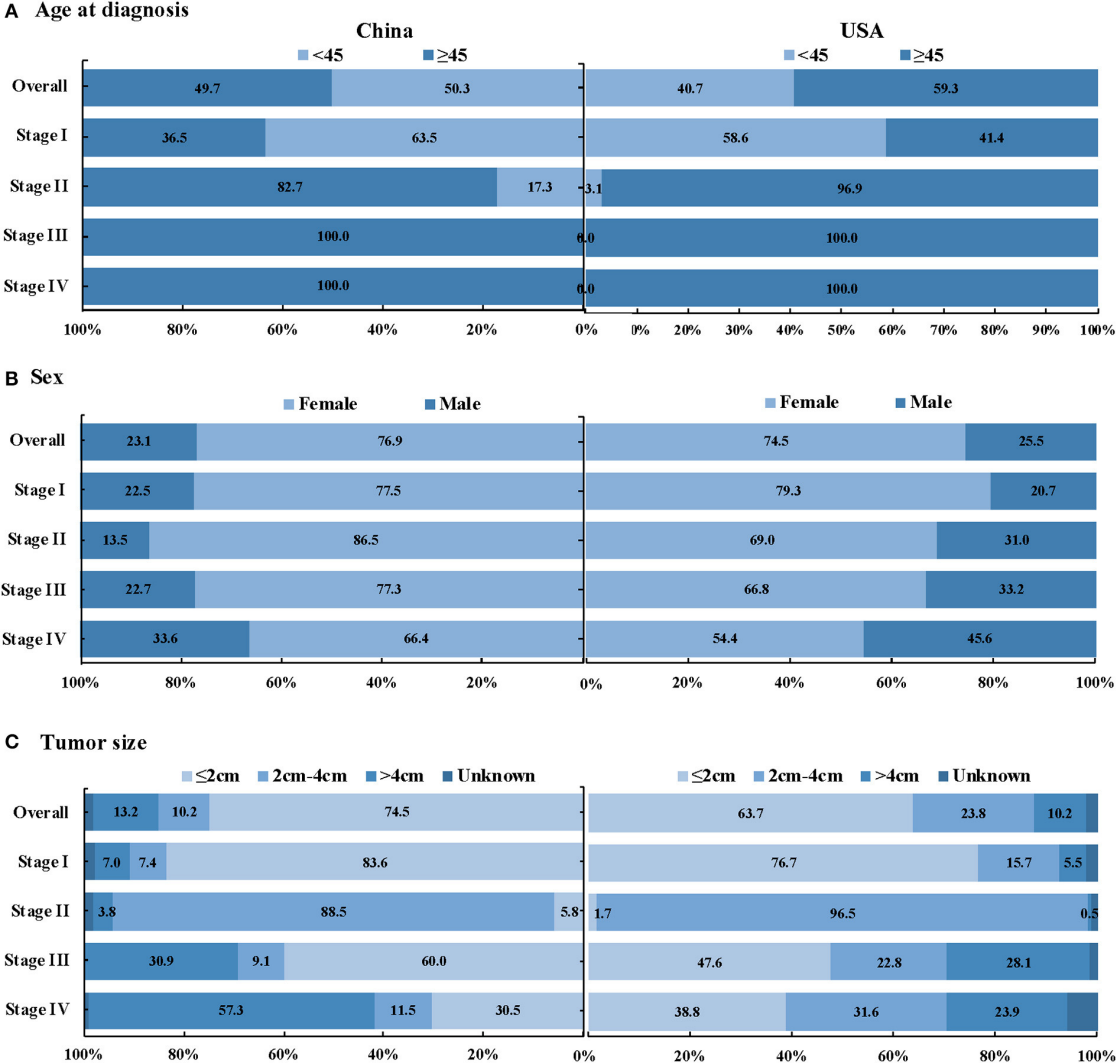
Stage distribution of DTC patients between the study in China and SEER database in the USA, by age at diagnosis, sex and tumor size **(A)** Age at diagnosis, **(B)** Sex, and **(C)** Tumor size.

As shown in Table 4, compared with the SEER database, our study had more early-stage DTC patients (81.2 vs. 76.0%, *P* < 0.001). The proportion of stage I in the study and SEER database in the USA were 78.4 and 69.0%, respectively. Besides, compared with the SEER database, we have higher proportion of DTC with 2 cm or smaller (*P* < 0.001), more TC patients aged < 45y (*P* < 0.001), higher proportion of lymph node metastasis (*P* < 0.001) and more unifocal tumors (*P* < 0.001). There were younger aged and < 2cm tumors in early-stage DTC patients in China than in the USA (*P* < 0.001) (Figure 2).

**Table 4:** Comparisons of clinicopathological features of DTC patients between the study in China and SEER database in the USA.

	**Study in China**	**SEER database in the USA**	***P*-value**
	***n* (%)**	***n* (%)**	
Sex			0.028
Male	431 (23.1)	2,802 (25.5)	
Female	1,437 (76.9)	8,204 (74.5)	
Age at diagnosis, year			< 0.001
< 45	940 (50.3)	4,479 (40.7)	
≥45	928 (49.7)	6,527 (59.3)	
Tumor size			< 0.001
≤ 2 cm	1,400 (74.9)	7,012 (63.7)	
2–4 cm	190 (10.2)	2,621 (23.8)	
>4 cm	247 (13.2)	1,122 (10.2)	
Unknown	31 (1.7)	251 (2.3)	
T stage			< 0.001
1	1,400 (74.9)	6,163 (56.0)	
2	190 (10.2)	1,918 (17.4)	
3	161 (8.6)	2,483 (22.6)	
4	86 (4.6)	337 (3.1)	
Unknown	31 (1.7)	105 (1.0)	
N stage			< 0.001
Absent	1,136 (60.8)	7,385 (67.1)	
Present	682 (36.5)	3,079 (28.0)	
Unknown	50 (2.7)	542 (4.9)	
M stage			0.025
Absent	1,850 (99.0)	10,803 (98.2)	
Present	17 (0.9)	188 (1.7)	
Unknown	1 (0.1)	15 (0.1)	
AJCC TNM staging system*			< 0.001
I	1,465 (78.4)	7,599 (69.0)	
II	52 (2.8)	768 (7.0)	
III	220 (11.8)	1,692 (15.4)	
IV	131 (7.0)	947 (8.6)	
Focality			< 0.001
Unifocal	1,210 (64.8)	6,472 (58.8)	
Multifocal	585 (31.3)	4,439 (40.3)	
Unknown	73 (3.9)	95 (0.9)	

^*^The Tumor-Node-Metastasis (TNM) staging system (7th edition) maintained by the American Joint Committee on Cancer (AJCC) was used for staging abstraction.

### Comparations of surgery and extent of surgery of DTC patients between the study and SEER database

The surgery rate of DTC patients of the study in China was slightly higher than that of SEER in the USA (99.9 vs. 97.2%, *P* < 0.001) (Table 5). There was no significant difference in the choice of surgery among stages in the study (*P* = 0.964), and almost all hospitalized patients underwent surgery in China. We found a significant difference of surgical treatment among stages in the USA (*P* < 0.001), and the surgical rate of stage IV was the lowest (90.4%). The surgical rate of stage IV was significantly higher than that in the USA (100.0 vs. 90.4%, *P* < 0.001). Besides, significant differences in surgery and extent of surgery were found among different regions in China (all *P* < 0.001)

**Table 5:** Comparisons on surgery of DTC patients between the study in China and SEER database in the USA.

	**Study in**		**SEER database**		**Study in China vs**.
	**China**		**in the USA**		**SEER database in the USA**
	***n* (%)**	***P-*value**	***n* (%)**	***P-*value**	***P-*value**
All	1,773 (99.9)	0.964	10,635 (97.2)	< 0.001	< 0.001
I	1,384 (99.9)		7,384 (97.8)		< 0.001
II	49 (100.0)		729 (95.7)		0.254
III	213 (100.0)		1,673 (99.1)		0.243
IV	127 (100.0)		849 (90.4)		< 0.001

Delete records: unknown stage, unknown surgery, unknown extent of surgery.

In Table 6, total thyroidectomy was more common than lobectomy both in the study and the SEER database (58.7 vs. 41.3% in the study; 83.0 vs. 17.0% in the SEER database). Significant differences were shown in surgical extensiveness (thyroid lobectomy vs. total thyroidectomy). With the aggravation of stage, the proportion of total thyroidectomy increased (*P* < 0.001). The proportion of total thyroidectomy at stage I, II, III and IV were 51.6, 55.1, 4.0, and 95.3% in our study, respectively (*P* < 0.001), and the corresponding proportion of total thyroidectomy at each stage were 79.6, 83.1, 91.3, and 96.2%, respectively (*P* < 0.001).

**Table 6:** Comparisons on extent of surgery of DTC patients between the study in China and SEER database in the USA.

	**Study**			**SEER database**			**Study in China vs. SEER**
	**in China**			**in the USA**			**database in the USA**
	**Lobectomy**	**Total thyroidectomy**	***P-*value**	**Lobectomy**	**Total thyroidectomy**	***P-*value**	***P-*value**
	***n* (%)**	***n* (%)**		***n* (%)**	***n* (%)**		
All	732 (41.3)	1,041 (58.7)	< 0.001	1,806 (16.5)	8,829 (80.7)	< 0.001	< 0.001
I	670 (48.4)	714 (51.6)		1,505 (20.4)	5,879 (79.6)		< 0.001
II	22 (44.9)	27 (55.1)		123 (16.9)	606 (83.1)		< 0.001
III	34 (16.0)	179 (84.0)		146 (8.7)	1,527 (91.3)		0.001
IV	6 (4.7)	121 (95.3)		32 (3.8)	817 (96.2)		0.604

Delete records: unknown stage, unknown surgery, unknown extent of surgery.

Besides, we found the proportion of total thyroidectomy in our study was significantly lower than that in the SEER database (58.7 vs. 80.7%, *P* < 0.001). Similar findings were observed in stage I-III (*P* < 0.001). No significant difference was observed in the extent of surgery in stage IV between China and the USA (*P* = 0.604).

## Discussion

To our knowledge, this was the first large-scale, multicentered study collecting updated and comprehensive clinicopathological and surgery information on DTC in China. Findings from the present study showed that the prevalence of late-stage DTC was 18.5%. Male, patients with NCMS had an increased risk of late-stage. Compared with the SEER database, more early-stage and young-aged DTC patients with smaller sizes were detected in China. The proportion of lobectomy in our database was significantly higher than that in the SEER database. Our findings gave us a better understanding of clinicopathological characteristics of hospitalized DTC patients in China and provided informative evidence for decision-making in DTC prevention and control.

Stage information is crucial for prognosis and survival (10–12). Compared with stage I DTC, the 10-year disease-specific survival of stage IV patients decreased by almost 27.9% (11). Over 80% of TC cases were diagnosed as early-stage in China, exceeding the USA (73.4%). The increasing use of ultrasonography for TC screening in routine check-ups in China might be one of the reasons for this phenomenon. However, it was not recommended in the USA (17). The proportion of early-stage in this study was also higher than that in South Korea in 2008 (53.8%) (18). However, the proportion of early-stage in the study (81.2%) was lower than that in a previous single-center analysis in northeast China (99.6%) and in eastern China (83.91%), and lower than that in a population-based cohort study in East Asia (84.4%) (19, 20). This difference could be due to this time trend analysis only covering one hospital, which may cause bias. Our study is the first large-scale, multicenter study in China, which could truly reflect the staging of DTC patients in grade A tertiary specialized hospitals in China.

In this multicentered study, the female/male sex ratio of DTC was 3.3, while male patients aged ≥45y had a higher risk of advanced DTC than females. According to the National Cancer Registry of China, the incidence rate of TC in women was 3.04 times that of men in 2015 (6). A similar female/male ratio (three-fold) was found in Global Cancer Statistics 2020 (1). The interaction between reproductive hormones in women, particularly estrogen, and the thyroid may lead to the generation of thyroid nodules (21, 22). However, autopsy evidence indicated that the actual incidence of thyroid nodules might not be much different between women and men (23). Since perinatal care and gynecological and obstetric clinics can provide women with more access to medical services, they may have additional opportunities to receive thyroid examinations (22, 24). This can harm both females who are subject to over-detection, and males who may be at risk of under-detection. In the study, although the numbers of males were fewer than females, male patients aged ≥45y had a higher risk of advanced DTC than females, in line with the existing evidence that DTC presented more advanced stage, higher mortality and recurrence rates in males than in females (25–27).

We found that access to NCMS was a risk factor for advanced DTC. Patients living in urban areas have a higher detection rate, but patients with NCMS were more likely to be diagnosed as late-stage (28). Considering the medical insurance system in China, this risk factor largely reflected the disease difference between urban and rural areas (29). Similar results were found in the latest study covering five major cancers in China, which showed a persistent diagnostic disparity that patients with NCMS had a higher risk of late-stage diagnosis compared with patients with urban insurance (OR = 1.4) (30). This phenomenon may be related to urban-rural inequities in medical utilization. Patients with urban medical insurance have better socioeconomic conditions and more access to medical resources, screening and other healthcare services (31, 32). Besides, compared with rural residents with NCMS, urban dwellers with urban medical insurance might also have a higher awareness of cancer prevention and regular doctor appointment in China (33, 34). Geographic disparities were also found in a prior report (30, 31, 35). Urban-rural disparities reflected that the current allocation of medical resources in China may be inequitable (36). This inequity should be considered in future TC prevention and control policy-making.

More young-aged, early-staged and small-sized TC patients were detected in our database compared with the SEER database. A population-based study proved that the incidence of young/middle-aged TC was on the rise in high-resource countries (37). Studies in South Korea and USA showed that the increased detection rate of early-stage and microcarcinoma may be related to the over-diagnosis caused by ultrasound screening (21, 22, 38–40). After TC screening was stopped, the proportion of localized stage of TC in South Korea declined gradually from 2005 to 2016 (39, 41). Previous studies indicated that the estimated proportion of TC cases attributable to overdiagnosis accounted for approximately 83% in women in China between 2008 and 2012 (5, 21). The high proportion of early-stage and small size tumors in our study also implied a warning signal of TC overdiagnosis caused by inappropriate ultrasonography screening for asymptomatic persons in China (5, 25, 42, 43). However, the acceleration of China's urbanization, the increasing access to routine physical examination, the improvement of diagnostic sensitivity and accuracy and the improvement of people's health awareness can also contribute to the specific clinicopathological characteristics of TC patients in this study (5, 24, 38, 44).

With the international attention to over-diagnosis and accumulation of evidence, many updated international recommendations recommend against screening for TC in asymptomatic adults using either neck palpation or ultrasound (Supplementary Table S5) (17, 45). Korea also called to stop TC screening in 2014 in order to turn the epidemic tide. The operation rate decreased significantly after TC screening was stopped in Korea (39). And the 5-year relative survival rate increased from 94.2% in 1993–1995 to 100.3% in 2011–2015 (46). Experience with TC screening in South Korea could serve as a cautionary tale for China. To date, there is no national TC screening plan in China. Only two provincial and municipal recommendations (Shanghai and Chongqing) on screening and prevention of common malignant tumors for residents, recommend thyroid-related examinations (clinical neck physical examination or ultrasonography) regularly for the general population. The wide application of ultrasound screening in China leads to the high detection of early-stage and small tumors (42). Evidence on the effectiveness of TC screening was at least insufficient if not disapproval. More considerations are needed for the necessity of TC screening in China. Considering the good prognosis of TC, large-scale and prospective studies are still needed to evaluate its long-term cost-effectiveness.

Subjects in this study were inpatients in oncology specialist hospitals, while patients in the SEER database were collected in cancer registries, including inpatients and outpatients, which may lead to a slightly higher surgery rate in China than in the USA. Before 2015, surgery for TC was the most preferred treatment. With the accumulation of evidence (47, 48), several international associations advocated active surveillance (AS) for small and low-risk TC instead of immediate surgery (Supplementary Table S6) (49, 50). The ATA 2015 Guidelines also represented a cost-effective strategy regarding AS and the extent of surgery (51). In accordance with international guidelines, the Chinese Society of Clinical Oncology also recommended active surveillance for low-risk papillary thyroid microcarcinoma patients in 2016 (52).

We found that more DTC patients with early-stage took lobectomy treatment in our database than in the SEER database, indicating that the choice of operation in China might be more conservative than that in the USA, which is in line with the updated guidelines. The high proportion of low-risk tumors with early-stage and small size in this study may result in more conservative surgery. Prior studies indicated that total thyroidectomy for the treatment of PTC measuring 1.0–4.0 cm does not result in a clinically significant improvement in disease-specific survival compared with lobectomy (53, 54). The health-related quality of life of DTC patients is not associated with the extent of surgery (55). With the accumulation of evidence, the extent of surgery of TC is constantly updated. We summarized global updated guidelines on surgery and the extent of surgery among DTC patients (Supplementary Table S6). The updated ATA guidelines promote surgical de-escalation by targeting low-risk patients (56). The global trend toward more partial thyroidectomy with a significant increase in the proportion of lobectomy (56). The number of unilateral thyroidectomies without neck dissection increased 2.56 times between 2007 and 2011 in Korea (57). There was a significant increase in the proportion of lobectomy over the last 10 years in France (2010–2019) (58). In addition, we observed increasing conservative surgery in the SEER database from 2015 to 2018 (Supplementary Figure S1). In recent years, surgery management in China gradually evolved from aggressive treatment to a more conservative approach. A Time trend analysis of TC surgery between 2008 and 2017 in a single institutional database of 15,000 Chinese patients showed that TC patients receiving total thyroidectomy decreased from 71 to 41% (59). A previous study showed that 53% of DTC with low to intermediate risk of recurrence took lobectomy in 2018–2019 (55). The proportion of lobectomy in early-stage DTC patients in our study was similar to previous studies. The decrease in total thyroidectomy indicated the benefits of evidence-based guidelines in accelerating changes to clinical practice to reduce overtreatment. Considering disparities in economic level, medical care level and surgical accessibility among regions, significant differences in stage and surgery extent among different regions.

Besides, we found no aggressive association between higher BMI and clinicopathological characteristics in our study. Similar to our results, previous studies have shown that a higher BMI is not associated with more aggressive tumor features (late-stage, lymph node metastasis, distant metastasis, etc.) (15, 60). In addition, a prior study showed that subjects in the underweight group had a significantly greater risk for distant metastasis (OR = 34.5) and macroPTCs (OR = 3.99) when compared with the normal-weight group (61). Paes et al. even indicated that a higher BMI was associated with a lower risk of tumor invasion and nodal metastases (62). On the contrary, several studies suggested that obesity was significantly related to larger tumor size, lymph node metastasis and advanced stage of PTC (aggressive features) (14, 63, 64). The causes remain uncertain. Biological factors, such as thyroid-stimulating hormone serum levels, have been considered contributors to increasing PTC aggressiveness in obese patients. TSH level has a positive correlation with vascular endothelial growth factor expression, and vascular endothelial growth factor can stimulate angiogenesis and increase vascular permeability (65). Cheng et al. found that leptin mediated the migration of PTC by activating the PI3K/AKT and MAPK pathways (66). Experimental models have shown that adipokines such as leptin and hepatocyte growth factor may regulate cancer cell proliferation and invasiveness (67).

## Strengths and limitations

This is a multicentered retrospective hospital-based study. Eight participating hospitals are distributed in 5 geographic regions of China, which enhances the representativeness of the study sample. What's more, since all TC patients are inpatients, detailed diagnostic and treatment information can be collected. However, several limitations of our study should be acknowledged. (1) Compared with the population-based SEER database, the sample size of our hospital-based database was limited, which cannot adequately represent current care in China. Even though, our multi-center hospital-based design took into consideration geographical variation, population density and socioeconomic status, which is the best available data at present. The extrapolation and interpretation of the differences should be cautious. (2) Information on height and weight was not available in individual hospitals, and the missing rate of BMI was relatively high (24.2%). This reduced the statistical power of tests, which might be the reason for the negative results between BMI and DTC stage. (3) Outpatients and non-hospitalized patients were not included, and the results may not be generalizable to the total population diagnosed and treated with TC during this period. (4) Affected by the geographical location, social and cultural background and other factors of China and the United States, the tumor biology may be different in the two countries, such as pathological type and stage. Further studies are warranted to confirm these findings and establish potential relationships between obesity and TC biology.

## Conclusions

This multicentered epidemiology study depicts clinicopathological features of DTC in China and compared them with the SEER database. Unique risk factors are found to be associated with late-stage DTC in China. The differences in clinicopathological features and surgical approaches between China and the USA indicate that the two countries' screening strategies and clinical practices are different. Overdiagnosis and overtreatment exist. Surgical comparisons provide clinical recommendations for examining physicians and surgeons. More research is needed for DTC prevention and control policy-making in China. These findings are intriguing, provided scientific evidence to clinical practice and research, were informative for screening strategy and treatment guidelines in China and provided references for other countries with similar patterns.

## Data availability statement

The raw data supporting the conclusions of this article will be made available by the authors, without undue reservation.

## Ethics statement

The study was conducted according to the guidelines of the Helsinki declaration and was approved by the Institutional Review Board of the National Cancer Center/National Clinical Research Center for Cancer/Cancer Hospital, the Chinese Academy of Medical Sciences, and Peking Union Medical College (Approval Number: 2019/146-1930). The patients/participants provided their written informed consent to participate in this study.

## Author contributions

JZ, KS, JW, SL, SC, and WW contributed to the conception and design of the study. YHe, DL, SL, YHu, MZ, BS, XL, HL, QZ, MS, LG, YZ, YLi, YLu, JT, YX, HS, HX, YJ, CY, JQ, HZ, RZ, and SZ contributed to data collection and quality control. JZ, KS, and JW drafted the paper and interpreted the results. All authors contributed to data interpretation and rewriting the paper, reviewed and approved the final version, and had full access to all the data and were responsible for the decision to submit the manuscript.

## Funding

The National Key Research and Development Program of China, Grant/Award Number: 2018YFC1311704.

## Conflict of interest

The authors declare that the research was conducted in the absence of any commercial or financial relationships that could be construed as a potential conflict of interest.

## Publisher's note

All claims expressed in this article are solely those of the authors and do not necessarily represent those of their affiliated organizations, or those of the publisher, the editors and the reviewers. Any product that may be evaluated in this article, or claim that may be made by its manufacturer, is not guaranteed or endorsed by the publisher.
