# Supplementary figures and images for: Clinicopathological and surgical comparisons of differentiated thyroid cancer between China and the USA: A multicentered hospital-based study
Source: Front Public Health. 2022 Sep 28;10:974359. doi: 10.3389/fpubh.2022.974359 (PMC9554273; doi:10.3389/fpubh.2022.974359)

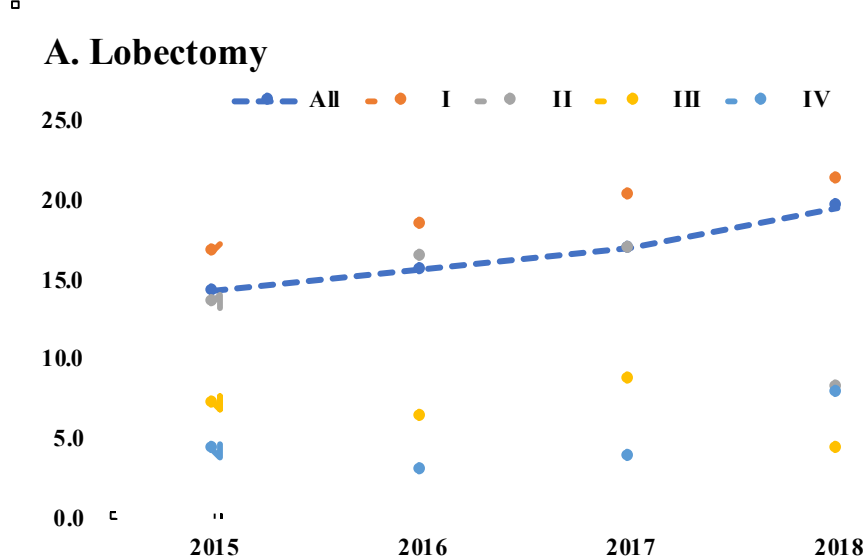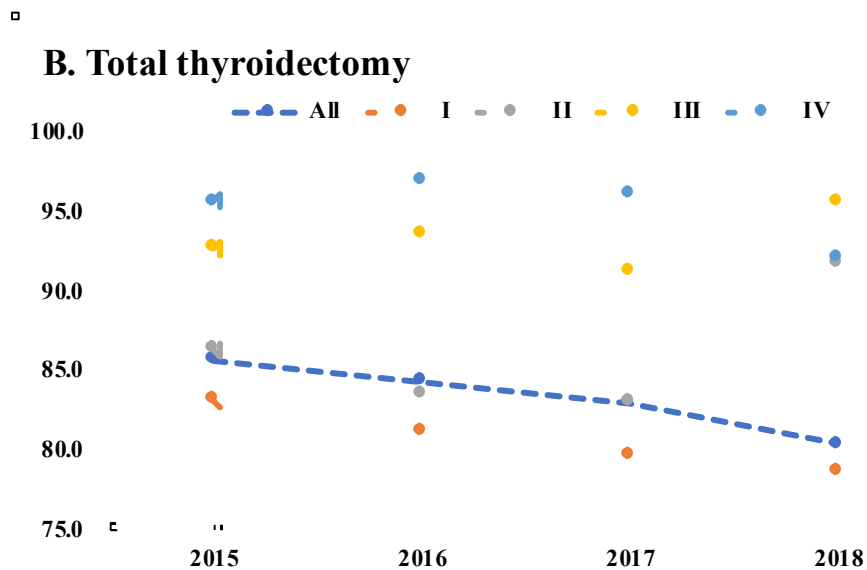

**Figure S1 Changes on extent of surgery of DTC patients in the USA, from 2015-2018**

Supplement: Supplementary file 1 [file Data_Sheet_1.PDF]
