# Supplementary material for: Clinicopathological and surgical comparisons of differentiated thyroid cancer between China and the USA: A multicentered hospital-based study
Source: Front Public Health. 2022 Sep 28;10:974359. doi: 10.3389/fpubh.2022.974359 (PMC9554273; doi:10.3389/fpubh.2022.974359)
Supplement: Supplementary file 2 [file Data_Sheet_2.PDF]

Supplementary Table S1 Characteristics of all thyroid cancer patients

	Frequency (N)	Proportion (%)
All	1970	100.0
Area		
Urban	1046	53.1
Rural	924	46.9
Age at diagnosis, year		
Mean ± SD	44.3±11.5	
<45	977	49.6
≥45	993	50.4
Sex		
Male	462	23.5
Female	1508	76.5
BMI, kg/m^2		
Mean ± SD	23.9±3.6	
<25.0	989	50.2
≥25.0	504	25.6
Unknown	477	24.2
Medical insurance		
Urban medical insurance	621	31.5
NCMS	443	22.5
Others	864	43.8
Unknown	42	2.1
Smoking history		
Never	1770	89.8
Ever	188	9.5
Unknown	12	0.6
Drinking history		
Never	1842	93.5
Ever	106	5.4
Unknown	22	1.1
History of thyroid diseases		
Absent	1750	88.8
Present	214	10.9
Unknown	6	0.3
Family history of thyroid cancer		
Absent	1922	97.6
Present	20	1.0
Unknown	28	1.4

Supplementary Table S2 Characteristics of DTC patients by stage distribution, by age

	**Age < 45**			**Age ≥ 45**							
	**Stage I n (%)**	**Stage II n (%)**	***P value***	**Stage I n (%)**	**Stage II n (%)**	**Stage III n (%)**	**Stage IV n (%)**	***P value***	**Early-stage (I/II) n (%)**	**Late-stage (III/IV) n (%)**	***P value***
All	931 (99.0)	9 (1.0)		534 (57.5)	43 (4.6)	220 (23.7)	131 (14.1)		577 (62.2)	351 (37.8)	
Area			0.742					0.084			0.051
Urban	542 (58.2)	6 (66.7)		269 (50.4)	19 (44.2)	102 (46.4)	50 (38.2)		288 (49.9)	152 (43.3)	
Rural	389 (41.8)	3 (33.3)		265 (49.6)	24 (55.8)	118 (53.6)	81 (61.8)		289 (50.1)	199 (56.7)	
Sex			1.000					0.002			0.004
Male	227 (24.4)	2 (22.2)		103 (19.3)	5 (11.6)	50 (22.7)	44 (33.6)		108 (18.7)	94 (26.8)	
Female	704 (75.6)	7 (77.8)		431 (80.7)	38 (88.4)	170 (77.3)	87 (66.4)		469 (81.3)	257 (73.2)	
BMI, kg/m2			0.730					0.503			0.983
< 25.0	518 (69.5)	7 (77.8)		247 (63.0)	16 (50.0)	103 (60.9)	50 (64.1)		263 (62.0)	153 (61.9)	
≥ 25.0	227 (30.5)	2 (22.2)		145 (37.0)	16 (50.0)	66 (39.1)	28 (35.9)		161 (38.0)	94 (38.1)	
Medical insurance			0.614					<0.001			0.001
Urban medical insurance	305 (33.7)	2 (22.2)		181 (34.3)	9 (20.9)	64 (29.5)	17 (13.3)		189 (33.3)	81 (23.5)	
NCMS	182 (20.1)	1 (11.1)		117 (22.3)	11 (25.6)	62 (28.6)	50 (39.1)		128 (22.5)	112 (32.5)	
Others	417 (46.1)	6 (66.7)		228 (43.4)	23 (53.5)	91 (41.9)	61 (47.7)		251 (44.2)	152 (44.1)	
Smoking history								0.232			
Never	839 (90.8)	9 (100.0)	1.000	478 (89.7)	42 (97.7)	199 (90.5)	112 (86.8)		520 (90.3)	311 (89.1)	0.569
Ever	85 (9.2)	0 (0.0)		55 (10.3)	1 (2.3)	21 (9.5)	17 (13.2)		56 (9.7)	38 (10.9)	
Drinking history								0.167			
Never	869 (94.5)	9 (100.0)	1.000	503 (94.9)	43 (100.0)	206 (94.5)	117 (91.4)		546 (95.3)	323 (93.4)	0.210
Ever	51 (5.5)	0 (0.0)		27 (5.1)	0 (0.0)	12 (5.5)	11 (8.6)		27 (4.7)	23 (6.6)	
History of thyroid diseases								0.731			
Absent	834 (90.0)	9 (100.0)	0.611	468 (87.6)	40 (93.0)	191 (86.8)	115 (87.8)		508 (88.0)	306 (87.2)	0.698
Present	93 (10.0)	0 (0.0)		66 (12.4)	3 (7.0)	29 (13.2)	16 (12.2)		69 (12.0)	45 (12.8)	
Family history of thyroid cancer								0.219			1.000
Absent	910 (98.7)	8 (100.0)	1.000	524 (99.4)	42 (97.7)	213 (99.5)	126 (98.4)		566 (99.3)	339 (99.1)	
Present	12 (1.3)	0 (0.0)		3 (0.6)	1 (2.3)	1 (0.5)	2 (1.6)		4 (0.7)	3 (0.9)	

Supplementary Table S3 Clinicopathological features of DTC patients according to BMI group

	All			Age < 45			Age ≥ 45			Male			Female		
	BMI < 25.0	BMI ≥ 25.0	*P value*	BMI < 25.0	BMI ≥ 25.0	*P value*	BMI < 25.0	BMI ≥ 25.0	*P value*	BMI < 25.0	BMI ≥ 25.0	*P value*	BMI < 25.0	BMI ≥ 25.0	*P value*
Tumor size			0.626			0.869			0.320			0.368			0.566
≤2cm	728 (79.1)	376 (78.0)		391 (77.4)	177 (78.0)		337 (81.2)	199 (78.0)		108 (70.6)	115 (75.2)		620 (81.8)	261 (79.3)	
>2cm	192 (20.9)	106 (22.0)		114 (22.6)	50 (22.0)		78 (18.8)	56 (22.0)		45 (29.4)	38 (24.8)		147 (19.2)	68 (20.7)	
T stage			0.534			0.387			0.779			0.388			0.598
1	728 (79.1)	376 (78.0)		391 (77.4)	177 (78.0)		337 (81.2)	199 (78.0)		108 (70.6)	115 (75.2)		620 (80.8)	261 (79.3)	
2	104 (11.3)	60 (12.4)		67 (13.3)	32 (14.1)		37 (8.9)	28 (11.0)		25 (16.3)	18 (11.8)		79 (10.3)	42 (12.8)	
3	60 (6.5)	26 (5.4)		36 (7.1)	10 (4.4)		24 (5.8)	16 (6.3)		14 (9.2)	10 (6.5)		46 (6.0)	16 (4.9)	
4	28 (3.0)	20 (4.1)		11 (2.2)	8 (3.5)		17 (4.1)	12 (4.7)		6 (3.9)	10 (6.5)		22 (2.9)	10 (3.0)	
N stage			0.427			0.441			0.713			0.719			0.067
Absent	525 (57.6)	281 (59.8)		246 (49.4)	114 (52.5)		279 (67.4)	167 (66.0)		66 (43.1)	66 (45.2)		459 (60.5)	215 (66.4)	
Present	387 (42.4)	189 (40.2)		252 (50.6)	103 (47.5)		135 (32.6)	86 (34.0)		87 (56.9)	80 (54.8)		300 (39.5)	109 (33.6)	
M stage			0.445			0.738			0.446			0.971			0.490
Absent	928 (98.7)	480 (99.2)		518 (98.9)	227 (99.1)		410 (98.6)	253 (99.2)		160 (99.4)	152 (99.3)		768 (98.6)	328 (99.1)	
Present	12 (1.3)	4 (0.8)		6 (1.1)	2 (0.9)		6 (1.4)	2 (0.8)		1 (0.6)	1 (0.7)		11 (1.4)	3 (0.9)	
AJCC TNM staging system			0.197			0.593			0.503			0.846			0.193
I	765 (81.3)	372 (76.9)		518 (98.7)	227 (99.1)		247 (59.4)	145 (56.9)		127 (78.9)	115 (75.2)		638 (81.8)	257 (77.6)	
II	23 (2.4)	18 (3.7)		7 (1.3)	2 (0.9)		16 (3.8)	16 (6.3)		3 (1.9)	3 (2.0)		20 (2.6)	15 (4.5)	
III	103 (11.9)	66 (13.6)		-	-		103 (24.8)	66 (25.9)		17 (10.6)	21 (13.7)		86 (11.0)	45 (13.6)	
IV	50 (5.3)	28 (5.8)		-	-		50 (12.0)	28 (11.0)		14 (8.7)	14 (9.2)		36 (4.6)	14 (4.2)	
AJCC TNM staging system			0.135						0.983			0.431			0.367
I+II	788 (83.7)	390 (80.6)		-	-	-	263 (63.2)	161 (63.1)		130 (80.7)	118 (77.1)		658 (84.4)	272 (82.2)	
III+IV	153 (16.3)	94 (19.4)		-	-	-	153 (36.8)	94 (36.9)		31 (19.3)	35 (22.9)		122 (15.6)	59 (17.8)	

Supplementary Table S4 Comparisons of the 7th and 8th editions of the AJCC TNM staging systems of DTC patients

	**AJCC TNM staging system (7th edition)**	**AJCC TNM staging system (8th edition)**	***P value***
I	1465 (78.4)	1736 (92.9)	<0.001
II	52 (2.8)	92 (4.9)	
III	220 (11.8)	31 (1.7)	
IV	131 (7.0)	9 (0.5)	

Supplementary Table S5 Recommendations on thyroid cancer screening in asymptomatic persons

Country	Year	Institution	Recommendations
**China**	There is no national screening plan on thyroid cancer in China		
	2021	Provincial and municipal level: recommendation on screening and prevention of common malignant tumors for residents (2020 Edition) issued by Shanghai anti-cancer association and Cancer Hospital Affiliated to Fudan University	1. Thyroid screening should be accompanied by functional examination and morphological examination<br>2. General population: clinical neck physical examination: once every 2-3 years at the age of 20-29 and once every year after the age of 30. Neck ultrasonography: once a year after the age of 30<br>3. High risk population: ultrasound examination once a year
	2020	Provincial and municipal level: guidelines for screening and prevention of common malignant tumors among Chongqing residents (2020 Edition)	1. Perform thyroid related examinations regularly.<br>2. High risk groups shall be rechecked in March, half a year and one year according to the specific situation.
**Canadian**	2015	The Canadian Task Force on the Periodic Health Examination	2015 Preventive Care Checklist Form not include examination of the thyroid
**Korea**	1999	The government initiated a national screening program for cancer and other common diseases	Although thyroid-cancer screening was not included in the program, providers frequently chose to offer screening with ultrasonography as an inexpensive add-on for $30 to $50.
	2014	A physician coalition for Prevention of Overdiagnosis of Thyroid Cancer	Thyroid cancer screening with ultrasonography be discouraged
**USA**	1996	American of Family Physicians	Recommended against screening for thyroid cancer using neck palpation or ultrasound in asymptomatic persons
	1996	US Preventive Services Task Force (USPSTF)	1.Recommended against screening for thyroid cancer in asymptomatic adults using either neck palpation or ultrasound (D recommendation)<br>2. C recommendation for screening in asymptomatic adults with a history of radiation of the external upper body (primarily the head and neck) in infancy or childhood
	2015	American Thyroid Association (ATA)	No recommendation
	2016	American Association of Clinical Endocrinologists (AACE), American College of Endocrinology (ACE), and Associazione Medici Endocrinologi (AME) Task Force	No recommendation
	2017	American Cancer Society (ACS)	No recommended screening test to find thyroid cancer early for people at average risk
	2017	US Preventive Services Task Force (USPSTF)	Recommends against screening for thyroid cancer in asymptomatic adults. (D recommendation)

Sort by alphabetical order of country first (A-Z), then by year

Supplementary Table S6 Global guidelines on surgery of DTC patients

Country	Year	Association	Updated Guideline	Recommendations on surgery and extent of surgery
**Canada**	2017	Cancer Care Ontario (CCO)	Cancer Care Ontario Thyroid Cancer Guideline: An Endorsement of the 2015 American Thyroid Association Management Guidelines for Adult Patients with Thyroid Nodules and Differentiated Thyroid Cancer	DTC<br>1.Thyroid lobectomy alone is sufficient treatment for small(≤1cm), unifocal, intrathyroidal carcinomas in the absence of prior head and neck radiation, familial thyroid carcinoma, or clinically detectable cervical nodal metastases.<br>2.For patients with thyroid cancer >1 cm and <4 cm without extrathyroidal extension, and without clinical evidence of any lymph node metastases (cN0), the initial surgical procedure can be either a bilateral procedure (near total or total thyroidectomy) or a unilateral procedure (lobectomy)<br>3.For patients with thyroid cancer >4 cm, or with gross extrathyroidal extension (clinical T4), or clinically apparent metastatic disease to nodes (clinical N1) or distant sites (clinical M1), the initial surgical procedure should include a near-total or total thyroidectomy<br>4.The treatment team may choose total thyroidectomy to enable RAI therapy or to enhance follow-up based upon disease features and/or patient preferences.
**China**	2016	Thyroid Cancer Professional Committee of the China Anti-cancer Association	Chinese Expert Consensus on Diagnosis and Treatment of Papillary Thyroid Microcarcinoma (2016)	PTMC:<br>1.Active Surveillance: low-risk PTMC patients (≤5mm, with no evidence of extracapsular extension, and no lymph node metastases, and no distant metastases, and no pathological high-risk subtypes, and no radiation exposure in childhood or adolescence, and no family history of thyroid cancer, and taking patients' preferences into consideration.<br>2.Lobectomy: unifocal, and unilateral, and intrathyroidal with no evidence of lymph node metastases, and no distant metastases, and no radiation exposure in childhood or adolescence, and no family history of thyroid cancer, and no nodule in the contralateral gland lobe, and low-risk recurrence<br>3.Total thyroidectomy: radiation exposure in childhood or adolescence, or family history of thyroid cancer, or extracapsular extension or lymph node metastases or distant metastases, or multifocal, or bilateral
**China**	2018	National Health Commission of the People's Republic of China	Thyroid Cancer Diagnosis and Treatment Norms (2018 Edition)	DTC:<br>1. T1 or T2, and unifocal, and without lymph node metastases and distant metastases, and in the absence of prior head and neck radiation, familial thyroid carcinoma, thyroid lobectomy was recommended<br>2. T3 or T4, total thyroidectomy was recommended
**China**	2021	Chinese Society of Clinical Oncology (CSCO)	Guides of Chinese Society of Clinical Oncology: Differentiated Thyroid Cancer 2021	DTC:<br>1. 1-4cm, no extrathyroidal extension, and no clinical evidence of any lymph node metastases (cN0), and no distant metastases, and no history of radiation exposure in head and neck area, thyroid lobectomy was recommended<br>2. >4cm, or extrathyroidal extension, or clinical lymph node metastases, or distant metastases, or poorly differentiated pathological subtypes, or patients with familial thyroid carcinoma or history of radiation exposure in head and neck area , total thyroidectomy was recommended
**Europe**	2019	European Society for Medical Oncology (ESMO) Guidelines Committee	Thyroid cancer: ESMO Clinical Practice Guidelines for diagnosis, treatment and follow-up	DTC:<br>1. Active surveillance can be proposed for unifocal papillary microcarcinomas (≤1 cm) with no evidence of extracapsular extension and lymph node metastases, and no radiation exposure in childhood or adolescence, and no family history of thyroid cancer, and not posterior or adjacent to trachea, and no aggressive features on cytology.<br>2. Lobectomy may be proposed for selected low-risk(T1a-T1b-T2, N0) tumors: ①T1aN0, and posterior or adjacent to trachea;②T1bN0, or T2N0, and unifocality, and no radiation exposure in childhood or adolescence, and no family history of thyroid cancer, and not posterior or adjacent to trachea, and no aggressive features on cytology<br>3. Total thyroidectomy: ①T1-T2N0, and (multifocality or radiation exposure in childhood or adolescence, or family history of thyroid cancer, or aggressive features on cytology); ②T3-T4N0; ③N1
**Italia**	2018	Six scientific Italian societies (the Italian Thyroid Association, the Medical Endocrinology Association, the Italian Society of Endocrinology, the Italian Association of Nuclear Medicine and Molecular Imaging, the Italian Society of	Italian consensus on diagnosis and treatment of differentiated thyroid cancer: joint statements of six Italian societies	DTC<br>1. Active surveillance(close follow-up) may be considered for very low-risk PTMC(intrathyroidal, with no clinical evidence of extra thyroid spread or metastases) the following setting:①patients at high surgical risk(elderly patients with incidentally discovered papillary cancer and have no evidence of extra thyroid spreading); ②patients who refuse surgical treatment; ③patients willing to enter into controlled clinical trials. A personal decision making is recommended as well as an accurate discussion with the patient to explain pro and cons of the active surveillance

		Unified Endocrine Surgery and the Italian Society of Anatomic Pathology and Diagnostic Cytology)		vs. surgical treatment.<br>2. Lobectomy may be considered for cancer ≤ 1 cm, clinically limited to one lobe, with no evidence of extrathyroidal extension or metastatic disease to nodes (cN0b) or prior head and neck irradiation.<br>3.Total thyroidectomy is recommended with at least one of the following parameters: patients with differentiated thyroid carcinoma > 4 cm, clinically (or intraoperative) detected cervical nodal metastases, gross extrathyroidal extension or metastatic disease to distant sites<br>4.Lobectomy or total thyroidectomy may be proposed to patients with differentiated thyroid carcinoma > 1 and < 4 cm without clinical (or intraoperative) evidence of extrathyroidal extension and lymph node metastases (N0b)
**Japan**	2018	Japan Association of Endocrine Surgery	2018 Japanese Clinical Practice Guidelines for Thyroid Tumors	PTC:<br>1. T1aN0M0 or T1bN0M0, total thyroidectomy was not recommended<br>2. In moderate risk patients, total thyroidectomy or lobectomy should be determined based on prognostic factors and patients' preferences.<br>3. >4cm, or extrathyroidal extension, or diameter of lymph node metastasis>3cm, or distant metastases, near-total or total thyroidectomy was recommended
**Japan**	2021	Japan Association of Endocrine Surgery Task Force on Management for Papillary Thyroid Microcarcinoma	Indications and Strategy for Active Surveillance of Adult Low-Risk Papillary Thyroid Microcarcinoma: Consensus Statements from the Japan Association of Endocrine Surgery Task Force on Management for Papillary Thyroid Microcarcinoma	PTMC(T1N0M0):<br>1.Immediate Surgery: ① PTMCs with clinical lymph node metastasis, or distant metastasis, or recurrent laryngeal nerve paralysis due to carcinoma invasion, or protrusion into the tracheal lumen warrant immediate surgery;② Tumors suspected of aggressive subtypes on cytology are recommended for immediate surgery;③Immediate surgery is also recommended for tumors adherent to the trachea or located along the course of the recurrent laryngeal nerve<br>2.Active Surveillance
**Poland**	2018	Polish Endocrine Society, Polish Society of Oncology, Polish Thyroid Association, Polish Society of Pathologists, Society of Polish Surgeons, Polish Society of Surgical Oncology, Polish Society of Clinical Oncology, Polish Society of Radiation Oncology, Polish Society of Nuclear Medicine, Polish Society of Paediatric Endocrinology, Polish Society of Paediatric Surgeons, Polish Society of Ultrasonography	Guidelines of Polish National Societies Diagnostics and Treatment of Thyroid Carcinoma. 2018 Update	1.Total/near total thyroidectomy is a basic surgical management.<br>2. Lobectomy with isthmus is acceptable in patients in whom papillary thyroid cancer was diagnosed in a single lesion ≤ 1 cm, cN0, if there are no indications for bilateral surgery and there is patient's consent for such management
**USA**	2015	American Thyroid Association (ATA)	2015 American Thyroid Association Management Guidelines for Adult Patients with Thyroid Nodules and Differentiated Thyroid Cancer	DTC:<br>1. An active surveillance management approach can be considered as an alternative to immediate surgery in patients with very low risk tumors (e.g., papillary microcarcinomas, <1cm, without clinically evident metastases or local invasion, and no convincing cytologic evidence of aggressive disease<br>2. Micropapillary thyroid cancers (<1 cm), and without extrathyroidal extension and clinical evidence of any lymph node metastases (cN0), and unifocal, and in the absence of prior head and neck radiation, familial thyroid carcinoma, the initial surgical procedure should be a thyroid lobectomy<br>3. 1-4cm, and without extrathyroidal extension and clinical evidence of any lymph node metastases (cN0), the initial surgical procedure should be a thyroid lobectomy, and total thyroidectomy depending on clinical risk factors, sonographic pattern, and patients' preferences.<br>4. >4 cm, or extrathyroidal extension, or lymph node metastases, or distant metastases, or patients with familial thyroid carcinoma or history of radiation exposure, the initial surgical procedure should be a near-total or total thyroidectomy
**USA**	2021	National Comprehensive Cancer Network (NCCN)	NCCN Clinical Practice Guidelines in Oncology-Thyroid carcinoma (Version 2.2021)	PTC:<br>1. <4cm, and no extrathyroidal extension, and no distant metastases, and no lateral cervical lymph node metastases, and no prior radiation therapy, lobectomy may be a treatment option in addition to thyroidectomy<br>2.>4cm, or extrathyroidal extension (grown beyond the thyroid into neck), or lymph node metastases, or distant metastases, or bilateral nodularity, or high-risk type of PTC, or radiation therapy in the neck area, a total thyroidectomy was recommended

Supplementary Table S7 STROBE and RECORD statement

	Item No.	STROBE items	Location in manuscript where items are reported	RECORD items	Location in manuscript where items are reported
**Title and abstract**					
	1	(a) Indicate the study's design with a commonly used term in the title or the abstract (b) Provide in the abstract an informative and balanced summary of what was done and what was found	(a) Title and Abstract<br>(b) Abstract	RECORD 1.1: The type of data used should be specified in the title or abstract. When possible, the name of the databases used should be included.<br>RECORD 1.2: If applicable, the geographic region and timeframe within which the study took place should be reported in the title or abstract.<br>RECORD 1.3: If linkage between databases was conducted for the study, this should be clearly stated in the title or abstract.	1.1 Abstract<br>1.2 Title, Abstract<br>1.3 Not applicable
**Introduction**					
Background rationale	2	Explain the scientific background and rationale for the investigation being reported	Introduction, Methods (Study design and participants)		
Objectives	3	State specific objectives, including any prespecified hypotheses	Introduction		
**Methods**					
Study Design	4	Present key elements of study design early in the paper	Methods (Study design and participants), Figure S1, Figure 1		
Setting	5	Describe the setting, locations, and relevant dates, including periods of recruitment, exposure, follow-up, and data collection	Methods (Study design and participants)		
Participants	6	(a) *Cohort study* - Give the eligibility criteria, and the sources and methods of selection of participants. Describe methods of follow-up<br>*Case-control study* - Give the eligibility criteria, and the sources and methods of case ascertainment and control selection. Give the rationale for the choice of cases and controls Cross-sectional study - Give the eligibility criteria, and the sources and methods of selection of participants<br>(b) *Cohort study* - For matched studies, give matching criteria and number of exposed and unexposed<br>*Case-control study* - For matched studies, give matching criteria and the number of controls per case	(a) Cross-sectional study-Methods (Study design and participants)<br>(b) Not applicable	RECORD 6.1: The methods of study population selection (such as codes or algorithms used to identify subjects) should be listed in detail. If this is not possible, an explanation should be provided.<br>RECORD 6.2: Any validation studies of the codes or algorithms used to select the population should be referenced. If validation was conducted for this study and not published elsewhere, detailed methods and results should be provided.<br>RECORD 6.3: If the study involved linkage of databases, consider use of a flow diagram or other graphical display to demonstrate the data linkage process, including the number of individuals with linked data at each stage	6.1 Methods (Study design and participants)<br>6.2 Not applicable<br>6.3 Not applicable
Variables	7	Clearly define all outcomes, exposures, predictors, potential confounders, and effect modifiers. Give diagnostic criteria, if applicable.	Methods (Study design and participants, outcome assessment)	7.1 Methods (Study design and participants, Staging abstraction), Statistical analysis	Methods (Study design and participants, outcome assessment)
Data sources/ measurement	8	For each variable of interest, give sources of data and details of methods of assessment (measurement). Describe comparability of assessment methods if there is more than one group	Methods (Study design and participants, statistical analysis）		
Bias	9	Describe any efforts to address potential sources of bias	Methods (Quality control, statistical analysis)		
Study size	10	Explain how the study size was arrived at	Methods (Study design and participants)		
Quantitative variables	11	Explain how quantitative variables were handled in the analyses. If applicable, describe which groupings were chosen, and why	Methods (Study design and participants, statistical analysis)		
Statistical methods	12	(a) Describe all statistical methods, including those used to control for confounding<br>(b) Describe any methods used to examine subgroups and interactions<br>(c) Explain how missing data were addressed<br>(d) *Cohort study* - If applicable, explain how loss to follow-up was addressed<br>*Case-control study* - If applicable, explain how matching of cases and controls was addressed<br>*Cross-sectional study* - If applicable, describe analytical methods taking account of sampling strategy<br>(e) Describe any sensitivity analyses	(a) Statistical analysis<br>(b) Flowchart and abstraction<br>(c) Statistical analysis<br>(d) Statistical analysis<br>(e) Statistical analysis		

Data access and cleaning methods				RECORD 12.1: Authors should describe the extent to which the investigators had access to the database population used to create the study population.<br>RECORD 12.2: Authors should provide information on the data cleaning methods used in the study.	12.1 Methods (Study design and participants)<br>12.2 Methods (Study design and participants, Quality control)
Linkage				RECORD 12.3: State whether the study included person-level, institutional-level, or other data linkage across two or more databases. The methods of linkage and methods of linkage quality evaluation should be provided.	12.3 Not applicable
**Results**					
Participants	13	(a) Report the numbers of individuals at each stage of the study (e.g., numbers potentially eligible, examined for eligibility, confirmed eligible, included in the study, completing follow-up, and analysed)<br>(b) Give reasons for non-participation at each stage.<br>(c) Consider use of a flow diagram	Results, Table S1, Table 1, Figure 1	RECORD 13.1: Describe in detail the selection of the persons included in the study (i.e., study population selection) including filtering based on data quality, data availability and linkage. The selection of included persons can be described in the text and/or by means of the study flow diagram.	13.1 Results, Figure 1
Descriptive data	14	(a) Give characteristics of study participants (e.g., demographic, clinical, social) and information on exposures and potential confounders<br>(b) Indicate the number of participants with missing data for each variable of interest<br>(c*) Cohort study* - summarise follow-up time (*e.g.,* average and total amount)	(a) Results, Table 1, Table S1, Table S2<br>(b) Table S1<br>(c) Not applicable		
Outcome data	15	*Cohort study* - Report numbers of outcome events or summary measures over time<br>Case-control study - Report numbers in each exposure category, or summary measures of exposure<br>*Cross-sectional study* - Report numbers of outcome events or summary measures	Results		
Main results	16	(a) Give unadjusted estimates and, if applicable, confounder-adjusted estimates and their precision (e.g., 95% confidence interval). Make clear which confounders were adjusted for and why they were included<br>(b) Report category boundaries when continuous variables were categorized<br>(c) If relevant, consider translating estimates of relative risk into absolute risk for a meaningful time period	Results, Table 2-6		
Other analyses	17	Report other analyses done—e.g., analyses of subgroups and interactions, and sensitivity analyses	Results, Table S3-S8, Figure S2		
**Discussion**					
Key results	18	Summarise key results with reference to study objectives	Discussion		
Limitations	19	Discuss limitations of the study, taking into account sources of potential bias or imprecision. Discuss both direction and magnitude of any potential bias	Discussion	RECORD 19.1: Discuss the implications of using data that were not created or collected to answer the specific research question(s). Include discussion of misclassification bias, unmeasured confounding, missing data, and changing eligibility over time, as they pertain to the study being reported.	Discussion
Interpretation	20	Give a cautious overall interpretation of results considering objectives, limitations, multiplicity of analyses, results from similar studies, and other relevant evidence	Discussion		
Generalisability	21	Discuss the generalisability (external validity) of the study results	Discussion		
**Other Information**					
Funding	22	Give the source of funding and the role of the funders for the present study and, if applicable, for the original study on which the present article is based	Acknowledgment		
Accessibility of protocol, raw data, and programming code				RECORD 22.1: Authors should provide information on how to access any supplemental information such as the study protocol, raw data, or programming code.	Data sharing
